# Colorectal Neoplasia in Inflammatory Bowel Disease

**DOI:** 10.3390/cancers17040665

**Published:** 2025-02-16

**Authors:** Eman Al Sulais, Turki AlAmeel, Maram Alenzi, Mohammad Shehab, Abdulelah AlMutairdi, Badr Al-Bawardy

**Affiliations:** 1 Department of Medicine, King Fahad Specialist Hospital, Dammam 32253, Saudi Arabia; ealslais@moh.gov.sa (E.A.S.);; 2 Department of Medicine, Division of Gastroenterology, Hepatology and Nutrition, Beth Israel Deaconess Medical Center, Harvard Medical School, Boston, MA 02215, USA; 3 Division of Gastroenterology, Department of Internal Medicine, Mubarak Alkabeer University Hospital, Kuwait University, Aljabreyah 47060, Kuwait; 4 Department of Internal Medicine, Division of Gastroenterology and Hepatology, King Faisal Specialist Hospital and Research Center, Riyadh 11121, Saudi Arabia; 5 College of Medicine, Alfaisal University, Riyadh 11533, Saudi Arabia; 6 Department of Internal Medicine, Section of Digestive Diseases, Yale School of Medicine, New Haven, CT 06510, USA

**Keywords:** inflammatory bowel disease, dysplasia, neoplasia, ulcerative colitis, Crohn’s disease

## Abstract

Patients with inflammatory bowel disease (IBD), including ulcerative colitis and colonic Crohn’s disease, are at an increased risk of developing colonic dysplasia and neoplasia. Multiple risk factors have been identified that increase the risk of colonic neoplasia in IBD, including but not limited to underlying disease extent, severity, duration, and concomitant primary sclerosing cholangitis. The overall risk of colonic neoplasia in IBD is decreasing but surveillance is still warranted in patients with high-risk features. In this review, we will discuss the epidemiology, pathogenesis, risk factors, approach to surveillance, and management of colonic neoplasia in IBD.

## Simple Summary

Patients with inflammatory bowel disease such as ulcerative colitis and colonic Crohn’s disease are at higher risk of developing colonic cancer. Overall, the risk of colon cancer in inflammatory bowel disease has been decreasing in the past decade likely owing to improved awareness, screening, and better control of inflammation. In this review, we outline the epidemiology, risk factors, screening, and management of colonic pre-cancerous lesions and colon cancer in inflammatory bowel disease.

## 1. Introduction

Inflammatory bowel diseases (IBDs), including Crohn’s disease (CD) and ulcerative colitis (UC), are autoimmune conditions characterized by relapsing and remitting intestinal inflammation. The highest prevalence of IBD is noted in Western regions, including North America and parts of Europe such as Scandinavia [1]. However, there continues to be a rise in IBD incidence rates in other regions including Asia, the Middle East, and Latin America [1,2]. Colorectal neoplasia (CRN) is the third most prevalent cancer and the second leading cause of cancer-related deaths in the United States. While the majority of CRN cases are sporadic, approximately 25% have a familial component, with 5% attributed to hereditary syndromes and others linked to pre-existing inflammatory bowel disease (IBD) [3].

Patients with IBD have a 1.5% to 3% greater risk of developing CRN compared to the general population [4]. It is estimated that IBD-related CRN accounts for 10–15% of the annual mortality among IBD patients [4]. Key risk factors for IBD-related CRN include primary sclerosing cholangitis (PSC), as well as the duration, extent, and severity of the colitis. Additional risk factors include colonic strictures, a history of dysplasia, and a family history of CRN [5].

In this review, we will discuss the epidemiology, pathogenesis, risk factors, and management of CRN in patients with IBD. Furthermore, we will examine the evidence supporting chemopreventive drug therapies and the role of surgery in managing CRN in patients with IBD.

## 2. Epidemiology of Colorectal Neoplasia in Inflammatory Bowel Disease

Colorectal cancer (CRC) is a leading cause of death in patients with IBD, historically accounting for approximately 15% of all-cause mortality in this population [6]. The link between IBD and CRC was first proposed by Burrill Crohn and Herman Rosenberg in 1925. In the early 1970s, a landmark study from Leeds, England, estimated the prevalence of CRC in IBD patients to be 5% at 10 years and 40% at 25 years following diagnosis [7,8].

Older epidemiological studies estimated the cumulative risk of dysplasia in patients with long-standing ulcerative colitis (UC) to be approximately 18% after 30 years [9]. More recent systematic reviews suggest a decrease in the incidence of CRN in IBD over the past decades. The incidence rate has decreased from 4.29 per 1000 patient-years in studies from the 1950s to 1.21 per 1000 patient-years in studies from the past decade [10]. This reduction may reflect improved disease management, surveillance, and early detection. This trend may also be due to inclusion of patients from non-tertiary care centers, which helps to mitigate referral bias [8]. A comparison of data from population-based studies versus referral center studies highlights a notable difference in CRC risk between the two groups. In a meta-analysis of population-based studies, the standardized incidence ratio (SIR) for CRC in IBD patients was 1.7 (95% CI, 1.2–2.2), while in four referral center studies, the SIR was significantly higher at 6.9 (95% CI, 4.1–9.7) [11].

A large population-based study conducted in Denmark and Sweden analyzed data from 96,000 patients with UC and compared them to 950,000 matched controls [12]. The researchers assessed CRC incidence and related mortality over a period spanning from 1969 to 2017. They found that the incidence of CRC in patients with UC was 1.29 per 1000 person-years, significantly higher than the 0.82 per 1000 person-years observed in the control group [Hazard Ratio (HR) 1.66, 95% CI 1.57–1.76]. Additionally, CRC-related mortality was elevated among UC patients compared to matched controls (HR 1.54, 95% CI 1.33–1.78). Notably, both the incidence and mortality rates of CRC in IBD patients declined in the last five years of the study (2013–2017), with an incidence rate of 1.38 and a reduced HR for CRC-related death of 1.25 [12].

In a comparable study conducted in Denmark and Sweden, approximately 47,000 patients with CD were compared to 470,000 matched controls over a 38-year period [13]. The incidence of CRC in patients with CD was found to be 0.82 per 1000 person-years, compared to 0.64 per 1000 person-years in the matched controls. This resulted in an overall adjusted HR of 1.40 (95% CI 1.27–1.53). Additionally, CRC-related mortality was higher among patients with CD, with rates of 0.47 per 1000 person-years compared to 0.31 per 1000 person-years in the control group. The risk of CRC was notably increased in patients with colonic CD (HR 1.76, 95% CI 1.46–2.11), while those with terminal ileitis did not show a significant increase in risk (HR 1.09, 95% CI 0.89–1.34) [13]. 

## 3. Pathogenesis of Colorectal Neoplasia in Inflammatory Bowel Disease

Patients with IBD are at an increased risk of developing CRN, with the carcinogenesis process driven by a complex interplay of factors including the host immune system, genetic predisposition, and the microbiome. Unlike sporadic CRN, which follows the adenoma–cancer pathway, IBD-related CRN progresses through a distinct inflammation-dysplasia-cancer sequence [14]. Chronic inflammation in IBD leads to oxidative stress-induced DNA damage, promoting the activation of tumor-promoting genes and inactivation of tumor-suppressor genes, further driving the development of cancer [15]. At the molecular level, the neoplastic pathway in IBD shares similar components with sporadic CRN, but their expression occurs at different stages. For example, P53 mutations/losses tend to be frequent and occur early on, even prior to the dysplasia–carcinoma sequence. Conversely, adenomatous polyposis coli (APC) gene mutations/loss is not common and occurs later in the dysplasia-to-carcinoma sequence in IBD [16]. Endoscopically, sporadic neoplasms usually present as few, distinct, visible lesions. However, IBD-related neoplasms may be undetectable by standard endoscopy and can spread across a larger surface area. Additionally, patients with IBD are at a greater risk of developing synchronous and metachronous neoplasms [17,18].

Dysbiosis plays a significant, though not fully understood, role in IBD-related CRN. The absence of certain bacteria, such as *Lactobacillus* and *Eubacterium aerofaciens*, due to colitis may be one contributing factor. An overabundance of carcinogenic bacteria is another key factor. Three bacterial species have been identified as carcinogenic in patients with IBD-related CRN: *Fusobacterium nucleatum*, *Escherichia coli* (polyketide synthetase (pks) positive), and *Bacteroides fragilis* expressing *B. fragilis* toxin [19]. Various mechanisms have been suggested to explain how these bacteria may contribute to the carcinogenesis process. One mechanism involves the impact of carcinogenic bacteria on cell wall antigens. For example, colibactin produced by *E. coli* can cause DNA double-strand breaks in intestinal cells, leading to chromosomal instability and subsequent cell transformation. Another potential mechanism is the induction of inflammation, and a third mechanism involves the production of carcinogenic substances, such as hydrogen sulfide [20].

## 4. Risk Factors of Colorectal Neoplasia in Inflammatory Bowel Disease

### 4.1. IBD Disease Severity

Endoscopic and histologic disease severity is a well-established risk factor for the development of IBD-related CRN [8]. Evaluating disease severity during a single surveillance session is insufficient for categorizing patients, as it is a dynamic process [15,21]. It has been demonstrated that histological evaluation may provide a more accurate method than endoscopic assessment for identifying patients at risk [22]. An exception is patients with PSC, who often exhibit minimal histological activity yet remain at a high risk of developing dysplasia. This necessitates their inclusion in an annual surveillance program, regardless of the disease course [15,22].

### 4.2. IBD Disease Extent

Patients with pancolitis exhibit a greater risk of IBD-related CRC compared to those with left-sided colitis. A pooled univariable analysis comparing extensive UC to left-sided disease demonstrated an OR of 2.43 (95% CI, 2.01–2.93; I^2^ = 0%) for the development of advanced CRN, based on data from 40 studies [23]. It is important to note that to accurately assess the extent of disease in IBD patients, histological evaluation is essential, as a reliance on endoscopic assessments may lead to an underrepresentation of the true extent of colitis [15]. 

Patients with UC who have had a J-pouch surgery demonstrate a remarkably low incidence of malignancy in the pouch, estimated at approximately 0.02% over a 20-year follow-up period [24]. On the contrary, patients who have undergone a colectomy continue to have a risk of rectal cancer. In a Danish population study from 1978 to 2018, the risk of cancer in the diverted rectum 10 years post colectomy in patients with IBD was significantly elevated with a HR of 7.56 (95% CI, 5.21, 10.86) compared to patients with IBD and without a colectomy [25]. This emphasizes the importance of continued surveillance of IBD patients with a diverted rectum.

### 4.3. IBD Disease Duration

A younger age at the time of IBD diagnosis, which typically indicates a longer disease duration, has been identified as a significant risk factor for the development of IBD-related CRC. In a large Danish cohort study involving 43,000 IBD patients, those diagnosed with UC before the age of 19 had a significantly higher relative risk (RR) of 43.8 (95% CI: 27.2–70.7) for developing CRC compared to patients diagnosed after the age of 40. Patients diagnosed between the ages of 20 and 39 had a lower but still elevated RR of 2.65 (95% CI: 1.97–3.56) [26]. Similar results were also noted in a population-based study of 96,000 patients in Denmark and Sweden. In that study, the HR for developing CRC was higher for patients with childhood-onset UC (HR 37.0, 95% CI 25.1–54.4), while those diagnosed with UC in later life showed no increased risk (HR 0.98, 95% CI 0.88–1.08) [12].

The risk of dysplasia and CRC remains closely tied to the duration of colitis. In a pre-biologic era study, Eaden and colleagues analyzed over 100 studies assessing CRC risk in IBD. They found a clear correlation between colitis duration and cancer risk. In the first decade of disease, the incidence rate of CRN was 2 per 1000 person-years (95% CI: 1–2/1000). In the second decade, the rate increased to 7 per 1000 person-years (95% CI: 4–12/1000), and by the third decade, it reached 12 per 1000 person-years (95% CI: 7–19/1000) [9].

### 4.4. Colonic Strictures

The European Crohn’s and Colitis Organization (ECCO) guidelines identify colonic strictures as a significant high-risk factor for the development of CRC in UC [27]. Colonic strictures are notably more prevalent in individuals with colonic CD, with approximately 10% of patients developing strictures in the colon. This rate is two to three times lower than the incidence of ileal strictures in the same population [28].

In a retrospective study conducted by the GETAID group, 248 patients with CD and 45 patients with UC underwent surgical resection of colonic strictures. Among the CD cohort, three (1%) were found to have low-grade dysplasia, one (0.4%) had high-grade dysplasia, and two (0.8%) were diagnosed with cancer. In the UC group, one (2%) had low-grade dysplasia, one (2%) had high-grade dysplasia, and two (5%) were found to have cancer. Notably, the absence of disease activity at the time of surgery was the only factor significantly associated with the presence of dysplasia or cancer at the stricture, with an OR of 4.86 (95% confidence interval, 1.11–21.27; *p* = 0.036) [29].

IBD-related CRC arising within the mucosa of a colonic stricture shares similar pathophysiological mechanisms with dysplasia in non-stricture segments. Furthermore, the presence of a stricture may serve as a surrogate marker for prior severe or chronic inflammation. However, the inability to traverse strictures raises concerns about the effectiveness of surveillance endoscopy and biopsy procedures [28]. Consequently, negative endoscopic mucosal biopsies taken from strictures do not effectively rule out the presence of dysplasia and in these cases referral for surgical evaluation is warranted.

### 4.5. Extensive Pseudopolyps (Post-Inflammatory Polyps)

Traditionally, the presence of extensive pseudopolyps has been thought to be associated with an increased risk of CRN, likely due to an impaired visibility of dysplastic/neoplastic lesions [30,31]. More recent studies, however, have not demonstrated a link between extensive pseudopolyps and a higher risk of CRN. In a retrospective multicenter cohort study of 1582 patients with IBD, the presence of pseudopolyps was associated with a higher rate of colectomy, but after a median follow-up period of 4.8 years, the rate of developing CRN was similar between those with and without pseudopolyps (HR 1.17; 95% CI: 0.59–2.31) [32]. A metanalysis of eight studies confirmed that the presence of pseudopolyps was not associated with a higher risk of CRN (multivariable HR, 1.73 [95% CI, 0.88–3.40]) [23]. Accordingly, the latest ECCO guidelines published in 2023 did not recommend shortening the interval of surveillance for patients with IBD based on the presence of pseudopolyps [5]. However, the presence of extensive pseudopolyps may hinder the quality of endoscopic surveillance and the limitation of optimal mucosal visibility should be considered when deciding on the surveillance interval.

### 4.6. Gender

Male patients with IBD carry a higher risk of developing CRN compared to female patients [33]. This result is consistent among studies. In a metanalysis, the estimated risk of developing CRN was 1.5-fold higher among the males, and in a different metanalysis the risk remained significant after multivariate regression analysis, with an OR of 1.27 [95% CI, 1.12–1.44] [23].

### 4.7. Primary Sclerosing Cholangitis

IBD associated with primary sclerosing cholangitis (PSC) is thought to represent a different phenotype of compared to IBD without PSC. Patients with IBD-PSC face a significantly higher risk of CRN compared to those with IBD alone. Specifically, the odds ratio for CRN in UC patients with PSC is 4.09 (95% CI, 2.89–5.76) compared to those with UC alone, while for CD patients with PSC, the odds ratio is 6.78 (95% CI, 1.65–27.9) compared to those with CD alone [34,35,36]. One theory suggests that the presence of secondary bile acids in the colon is carcinogenic, potentially contributing to the increased risk associated with coexisting PSC. These bile acids may promote inflammation and cellular changes that facilitate neoplastic transformation [37]. Lesions in patients with PSC tend to occur on the right side of the colon and often manifest early in the disease course, typically among younger patients [36,38]. Additionally, these individuals experience a progression rate to advanced neoplasia that is 3.4 times higher than that of non-PSC IBD patients [39]. Notably, the risk of colorectal neoplasia persists even after liver transplantation, with cumulative risks of 1.8% and 3.3% at 10 and 20 years post-transplant, respectively [40].

### 4.8. Smoking

Smoking is an established risk factor for developing sporadic CRN. However, its association with IBD-related CRN is not consistent among studies, and that might be related to its divergent effect on the course of disease in ulcerative colitis vs. Crohn’s disease. In a multicenter, prospective cohort of 576 patients with IBD (260 with CD) higher pack-year of smoking was associated with recurrent CRN in a dose dependent fashion [41]. However, in a large metanalysis, the pooled multivariate OR of smoking on CRN was not significant at 1.27 (95% CI, 0.75–2.13) [23].

### 4.9. Family History of CRC

A family history of CRC has been associated with an increased risk of IBD-related CRC. A meta-analysis encompassing 15 studies revealed that a family history of CRC correlates with a heightened risk of IBD-related CRC, yielding an OR of 2.62 (95% CI, 1.93–3.57; I^2^ = 0%) [23]. Table 1 summarizes some of the risk factors of CRN in IBD.

## 5. Endoscopic Surveillance Intervals and Modalities

Surveillance for dysplasia in patients with IBD utilizes various techniques and strategies aimed at the early detection and prevention of CRC. To maximize effectiveness, surveillance should be personalized based on the individual patient’s risk profile. These risk factors are broadly divided into two categories: potentially modifiable factors and non-modifiable factors [42].

Potentially modifiable risk factors for dysplasia in IBD include smoking, cumulative inflammatory burden, backwash ileitis (indicating more extensive colitis), post-inflammatory pseudopolyps, prior dysplasia, and the presence of a mass or stricture, all of which are associated with a heightened risk of neoplasia [32,42,43]. These factors often signify more severe inflammation and widespread disease involvement. In contrast, non-modifiable risk factors, such as male sex, longer disease duration, greater colonic involvement, family history of CRC (independent of IBD), the presence of PSC, and younger age at diagnosis (irrespective of disease duration), also contribute significantly to dysplasia risk [11,44,45,46].

In patients with UC and colonic CD, colonoscopy should be performed approximately 8 years following the onset of symptoms for dysplasia surveillance. For individuals with concurrent PSC, annual colonoscopy surveillance is warranted from the time of diagnosis, irrespective of disease activity or duration. If the initial surveillance colonoscopy is negative for dysplasia, then the interval of repeat surveillance colonoscopy is dependent on the patient’s risk profile.

**Figure 1:**
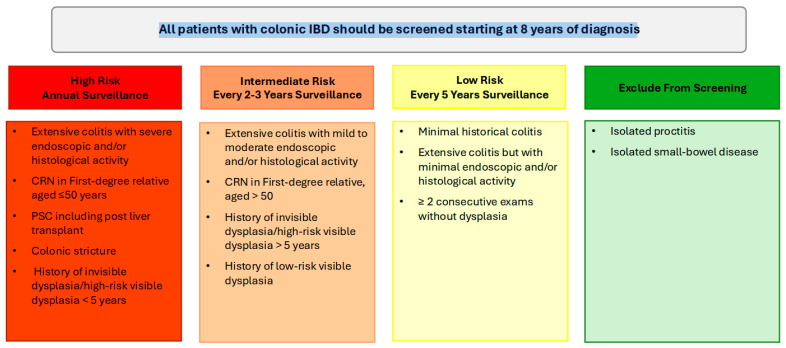
Summary of high, intermediate and low colorectal cancer risk profiles in inflammatory bowel disease and recommended surveillance colonoscopy intervals.

High-risk patients, including those with a family history of CRC (aged ≤ 50 years), colonic strictures, prior dysplasia, PSC, or extensive colitis with significant inflammation, should continue to undergo annual colonoscopy. Intermediate-risk patients should undergo screening every 2–3 years, while patients without high- or intermediate-risk factors can be screened every 5 years (Figure 1). Following a negative screening colonoscopy, the interval for subsequent surveillance should be tailored based on individual risk factors, including the extent of colonic inflammation, family history of CRC, presence of PSC, history of dysplasia, and the frequency and quality of prior examinations. Surveillance is most effective when the disease is in clinical and endoscopic remission, as this minimizes the likelihood of inflammation-related complications and allows for clearer detection of dysplastic changes [5]. Disease staging biopsies, obtained from multiple colonic segments, should be utilized to assess histologic disease activity and extent, which can guide future surveillance intervals [5,47].

### 5.1. Current Colorectal Neoplasia Surveillance Modalities

Several CRC surveillance modalities are commonly employed in IBD, with high-definition white-light colonoscopy (HD-WLC) recognized as the standard method. HD-WLC utilizes advanced high-definition imaging technology, which enhances the clarity of colonoscopy views and facilitates more accurate lesion detection. Evidence supporting this is provided by a study by Buchner et al., which revealed that the application of HD-WLC was independently associated with significantly higher dysplasia detection rates: the adenoma detection rate was 28.8% compared to 24.3% (*p* = 0.012) and the polyp detection rate was 42.2% versus 37.8% (*p* = 0.026) when contrasted with conventional standard-definition WLC. Although HD-WLC is widely available and demonstrates favorable overall detection rates, it is noteworthy that it may miss subtle lesions, particularly in areas characterized by active inflammation [48].

Non-targeted biopsy protocol refers to obtaining biopsies from four quadrants every 10 cm and had been historically standard practice during surveillance colonoscopies in IBD. However, non-targeted biopsies during HD-WLC demonstrated variability in effectiveness and yield for detecting dysplasia. This variability is highlighted by studies reporting contrasting results. For instance, Van den Broek et al. observed a notably low dysplasia detection rate of 0.2% from biopsies in a cohort of 1010 patients [49]. In contrast, Hu et al. reported a significantly higher dysplasia detection rate of 18.1% in a group of 442 patients with IBD, noting that 11.8% of dysplasia cases were identified exclusively through non-targeted biopsies [50]. With the advent of improved endoscopic imaging such as HD-WLC, it remains unclear if non-targeted biopsies are still essential to perform in all IBD surveillance colonoscopies. There have been multiple proposed patient groups in which non-targeted biopsies should be performed and include personal history of dysplasia, PSC, tubular colon, active inflammation, or when using standard definition colonoscopy [5,47].

Dye chromoendoscopy (DCE) is a technique that involves the topical application of dyes or pigments (methylene blue or indigo-carmine) to enhance the detection and delineation of surface abnormalities in the colon. By improving the visualization of mucosal patterns and accentuating subtle lesions, DCE is meant to enhance the overall effectiveness of surveillance and diagnosis in patients with IBD, serving as a valuable tool for the early detection and management of dysplasia and ultimately contributing to improved patient outcomes through timely intervention [51,52]. Performing DCE requires optimal bowel preparation and the absence of inflammation and is associated with longer procedure times.

A meta-analysis conducted by Subramanian et al. of six studies involving 1277 patients demonstrated that DCE yielded a 7% higher detection rate of dysplasia compared to WLC on a per-patient basis, with a 95% confidence interval of 3.2–11.3 and a number needed to treat (NNT) of 14.3 [53]. However, this metanalysis is outdated and included standard definition white light colonoscopy. The utility of DCE in the setting of HD-WLC remains controversial. A meta-analysis comparing virtual CE (VCE), DCE, and HD-WLC in 11 randomized controlled trials (RCTs) of 3205 patients did not demonstrate the superiority of DCE over HD-WLC [54]. However, an updated metanalysis of six RCTs involving 978 patients demonstrated superiority of DCE over HD-WLC with an OR 1.94 (95% CI 1.21–3.11, I^2^ = 28%, *p* = 0.006) in detecting more patients with dysplasia [55]. Table 2 summarizes findings of randomized controlled trials comparing HD-WLC to DCE in IBD. These findings underscore the value of DCE. It also highlights that DCE might not be needed in every surveillance colonoscopy in IBD and can potentially be reserved to be performed by experienced endoscopists in high-risk patients such as personal history of dysplasia and PSC.

In contrast, virtual chromoendoscopy (VCE) modalities, such as Narrow Band Imaging (NBI), represent advanced endoscopic techniques that utilize narrow wavelength spectrums of light to illuminate mucosal tissues. Unlike traditional WLC, NBI employs selective light filters to enhance visualization of the vascular and surface architecture of mucosal lesions without the need for dyes. While early studies did not demonstrate a significant advantage of VCE technologies for dysplasia detection in patients with IBD when using either standard-definition or HD-WLC, more recent investigations have indicated that HD NBI achieves dysplasia detection rates comparable to those of DCE or HD i-scan with DCE, with VCE methods also offering shorter withdrawal times [47]. A meta-analysis of eleven randomized controlled trials (RCTs) showed that VCE performed equally as well as both DCE and HD-WLC in terms of dysplasia detection on a per-patient basis [54].

### 5.2. Investigational Surveillance Modalities

Confocal Laser Endomicroscopy (CLE) represents a cutting-edge endoscopic technique that enables clinicians to acquire high-resolution images, facilitating a virtual histological examination of tissue. This advanced modality can be implemented in two primary configurations: through a microprobe that is introduced via the endoscope or through an endoscope that incorporates an integrated CLE function. By employing CLE, endoscopists can assess disease activity and intraepithelial neoplasia in real time, significantly enhancing diagnostic precision [64]. A pivotal study by Kiesslich et al. demonstrated the efficacy of combining CLE with Capsule Endoscopy (CE) for the detection of dysplasia in patients with ulcerative colitis (UC). This synergistic approach was shown to identify neoplasia at a rate 4.75 times greater than that achieved with conventional colonoscopy, thereby highlighting its potential to improve clinical outcomes for patients [65].

Autofluorescence imaging (AFI) is an innovative endoscopic technique that utilizes the natural properties of tissue fluorescence to improve the visualization of abnormalities. A study conducted by Broek et al. demonstrated that AFI significantly enhances the detection of neoplasia in patients with longstanding UC, achieving a remarkable 0% miss-rate compared to a 50% miss-rate observed with WLC. By color-coding neoplastic lesions in purple, AFI exhibits a high sensitivity of 100% for identifying neoplasia, thereby reducing the need for non-targeted biopsies [66].

Artificial intelligence (AI) has been validated as a reliable tool for enhanced adenoma detection during screening and surveillance colonoscopy in patients without IBD [67,68,69,70]. Recently, there has been a focused initiative to develop AI algorithms specifically for the detection and classification of dysplasia associated with IBD. Notably, Vinesard et al. introduced the IBD-CADe model, which was developed using a dataset of 1266 HD-WLC images and 426 dye-based chromoendoscopy images, all depicting histologically confirmed IBD-associated colorectal lesions. This model demonstrated a significant improvement in lesion detection during HD-WLC, achieving a sensitivity of 95.1%, specificity of 98.8%, positive predictive value (PPV) of 98.9%, negative predictive value (NPV) of 94.7%, accuracy of 96.8%, and an area under the curve (AUC) of 0.85 [71]. Looking forward, future advancements in this domain aim to integrate histological and endoscopic AI models into a cohesive platform. This integrated tool is expected to enhance disease monitoring and predictive capabilities, thereby offering a comprehensive approach to the management and detection of CRN in patients with IBD.

## 6. Dysplasia Definitions, Terminology and Management

Dysplasia is a well-established precursor to CRC and its early detection and management in patients with IBD are critical for CRC prevention. Current guidelines recommend routine surveillance in individuals with IBD, particularly those with extensive or long-standing disease, to facilitate the early identification and intervention of dysplastic lesions [64,72].

Dysplasia is characterized as either visible (detected on endoscopic examination) or invisible (detected only on non-targeted biopsies). Visible dysplasia is further characterized by morphology by the modified Paris classification as polypoid (≥2.5 mm in protrusion; sessile or pedunculated) and non-polypoid (<2.5 mm in protrusion; flat elevated, flat, flat depressed) [47,73]. Dysplasia in IBD is further classified into three categories by histopathology as: low-grade dysplasia (LGD), high-grade dysplasia (HGD), and indefinite dysplasia [47]. LGD is characterized by cells exhibiting mild abnormalities in size, shape, and organization, which indicates an increased risk of progression to HGD or CRC. LGD necessitates closer surveillance due to its potential for further development. In a single-center study, indefinite dysplasia has been shown to harbor increased risk of progression to advanced colorectal neoplasia necessitating close surveillance in this group also [74].

**Figure 2:**
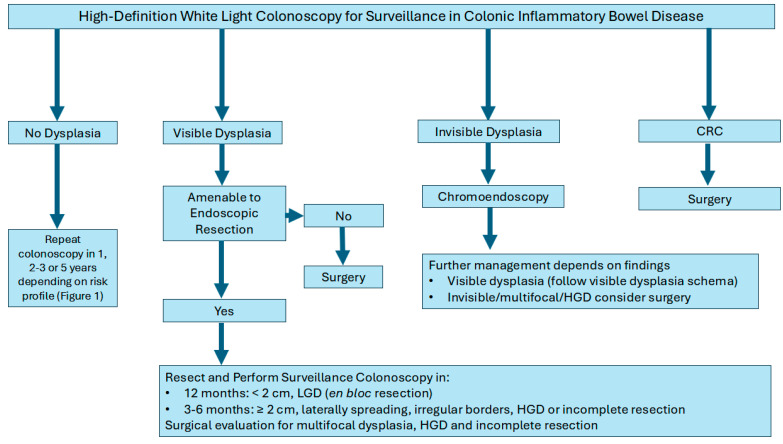
Algorithm for the management of colon cancer surveillance in inflammatory bowel disease.

Management strategies typically depend on the focality of the dysplasia (unifocal vs multifocal), endoscopic resection ability, and histopathology (LGD vs. HGD). For example, unifocal visible dysplastic lesions may be treated with endoscopic resection techniques such as endoscopic mucosal resection (EMR) or endoscopic submucosal dissection (ESD) followed by frequent colonoscopies to monitor for changes. Colectomy for example, may be considered for patients with multifocal dysplasia, lesion not amenable for full endoscopic resection or additional risk factors (PSC with dysplasia or CRC). Furthermore, if invisible dysplasia is detected on non-targeted biopsies and subsequently confirmed by an expert pathologist, it is imperative to conduct a repeat colonoscopy with chromoendoscopy. This procedure should include both targeted and non-targeted biopsies performed by an expert IBD endoscopist to ensure accurate assessment and management (Figure 2) [64,73].

Despite current surveillance practices, approximately 30% of CRC cases in patients can be classified as interval carcinomas as they were missed at previous colonoscopy [75]. In addition, up to half of the CRCs in patients who have had a colonoscopy in the past five years can be attributed to previously missed lesions despite adequate colonoscopy quality measures. This underscores the importance of optimizing surveillance techniques and approaches to reduce the risk of missed lesions and improve patient outcomes [72,76]. Surveillance colonoscopy is best performed in an adequately prepared colon, without significant inflammation and with an adequate colonoscopy withdrawal time. A recent study defined a colonoscopy withdrawal time of at least 15 minutes to be associated with visible dysplasia detection utilizing high-definition colonoscopy exams in IBD [77]. The study also demonstrated a 4.2% increase in visible dysplasia detection with every minute increase in colonoscopy withdrawal time (OR 1.04; 95% CI, 1.02–1.06; *p* = 0.001) [77].

Conversely, the effectiveness of existing surveillance guidelines has not been prospectively investigated in certain cases, which has resulted in the overutilization of healthcare resources, particularly given that the majority of IBD patients will not develop CRC. This highlights the pressing need for an evidence-based systematic approach to identify which IBD patients specifically require surveillance [78].

## 7. Chemoprevention of Colorectal Neoplasia in Inflammatory Bowel Disease

The disease duration, activity/severity, and extent of inflammation in IBD are well established risk factors for the development of CRC and dysplasia. Hence, therapeutic agents that are effective in induction and maintenance of remission in IBD should theoretically decrease the risk of CRC and dysplasia. It remains uncertain if individual agents have additional chemoprotective properties against CRC and dysplasia in IBD. Many of the studies examined the impact of individual therapeutic agents on the of risk dysplasia and CRC in IBD did not control for inflammation severity and duration.

(1). *5-aminosalicylic acid (5-ASA)*

The role of 5-aminosalicylic acid (5-ASA) in preventing CRC in patients with IBD is supported by several studies. The initial evidence emerged from a case-controlled study involving 102 patients with UC and CRC, matched to 196 controls without CRC. This study revealed that the use of 5-ASA, particularly sulfasalazine, was associated with a protective effect, yielding a relative risk (RR) of 0.38 (95% confidence interval [CI], 0.20–0.69) [79]. Another case-controlled study conducted within the CESAME (Cancers Et Surrisque Associé aux Maladies inflammatoires intestinales En France) cohort analyzed 114 patients with IBD who developed CRC, matched to 286 controls. This investigation demonstrated that aminosalicylates were protective from CRC, with an OR of 0.587 (95% CI: 0.367–0.937, *p* = 0.0257) [80].

In contrast, a systematic review and meta-analysis encompassing 26 observational studies with a total of 15,460 patients produced mixed results. While the analysis indicated that 5-ASA was protective against CRC, it did not demonstrate a protective effect against dysplasia. Notably, the protective effect of 5-ASA was significant among patients with UC (OR = 0.46, 95% CI: 0.34−0.61) but was not observed in those with CD. Additionally, the results suggested that mesalazine was protective, while sulfasalazine did not exhibit a similar benefit [81]. Another systematic review and metanalysis of 31 observational studies confirmed the chemoprotective role of 5-ASA in IBD, with evidence of a dose-dependent effect [82].

(2). *Thiopurines*

In a French prospective cohort study of 19,486 patients, thiopurine therapy was associated with a CRC protective effect with an aHR of 0.28 (95% CI: 0.1–0.9; *p* = 0.03) [83]. Supporting these findings, a systematic review and meta-analysis of 24 observational studies comprising 76,999 patients demonstrated that thiopurine use was associated with a reduced risk of developing CRC, with an OR of 0.63 (95% CI: 0.46–0.86). Notably, this protective effect was noted in patients with UC with an OR of 0.67 (95% CI: 0.45–0.98) and not CD with an OR of 1.06 (95% CI: 0.54–2.09) [84].

Furthermore, another meta-analysis encompassing 27 observational studies—comprising 16 case-control studies and 11 cohort studies—investigated a total of 95,397 patients. This analysis corroborated the protective effect of thiopurines against CRC, reporting an OR of 0.49 (95% CI: 0.34–0.70) for case-control studies and a relative risk (RR) of 0.96 (95% CI: 0.94–0.98) for cohort studies [85].

(3). *Biologics*

In a comprehensive cohort study involving a total of 62,007,510 patients with IBD, researchers investigated the potential of anti-tumor necrosis factor (anti-TNF) agents as chemopreventive agents for CRC. The results demonstrated that the use of anti-TNF agents was significantly associated with a reduced risk of developing CRC. Specifically, patients with CD exhibited an OR of 0.69 (95% CI: 0.66–0.73; *p* < 0.0001), while those with UC had an OR of 0.78 (95% CI: 0.73–0.83; *p* < 0.0001) [86]. These findings highlight the potential role of anti-TNF therapy in mitigating CRC risk among IBD patients. Use of biologics has also been identified as an independent risk factor with lower risk of advanced-IBD associated intestinal cancer in UC (not CD) in a nationwide study in Japan [87].

(4). *Statins*

Statins, which are hydroxymethylglutaryl coenzyme A reductase inhibitors, possess anti-inflammatory and antiproliferative properties in addition to their lipid-lowering effects. In a retrospective cohort study involving 11,000 patients (1376 of whom were on statins), it was found through propensity score matching that statin use was associated with a significant protective effect against developing CRC (OR: 0.42; 95% CI, 0.28–0.62) over a follow-up period of nine years in IBD [88]. Supporting these findings, a recent Swedish nationwide study matched 5273 statin users with an equal number of non-users, with a median follow-up of nine years. Statin use not only reduced the risk of CRC but also lowered CRC-related mortality in patients with IBD, with the protective effect being duration-dependent; patients on statins for two or more years had a lower likelihood of developing CRC [89].

(5). *Ursodeoxycholic acid (UDCA)*

A systematic review and meta-analysis of seven observational studies, including 707 patients with IBD using UDCA, found that UDCA was not associated with a reduced risk of developing CRC (RR = 0.87; 95% CI, 0.51–1.49; *p* = 0.62) [90].

(6). *Folic acid*

A systematic review and meta-analysis of ten studies, comprising 4517 patients, demonstrated that folic acid supplementation provided protective effects against CRC in patients with IBD (HR = 0.58; 95% CI, 0.37–0.80) [91].

(7). *Aspirin and Non-Steroidal Anti-Inflammatory Drugs*

A systematic review and meta-analysis of eight studies involving 14,917 patients on aspirin and three studies involving 1282 patients on NSAIDs found that neither aspirin nor NSAIDs had a protective role against CRC in patients with IBD, with ORs of 0.80 (95% CI: 0.39–1.21) and 0.66 (95% CI: 0.06–1.39), respectively [92].

## 8. Surgical Management of Colorectal Cancer and Dysplasia in Inflammatory Bowel Disease

Recent epidemiological studies suggest that, despite a decreasing incidence of surgery in UC, colorectal dysplasia and malignancy have become increasingly common indications for colectomy in this population [93]. The main indications for surgery include invisible dysplasia, visible dysplasia not amenable to endoscopic resection (no defined borders or due to submucosal invasion, and patients at high risk of malignancy, such as those with HGD and concomitant PSC [94].

Moreover, the presence of underlying IBD has been shown to negatively affect surgical outcomes in colorectal cancer patients. A large population-based study analyzing 400,000 patients who underwent CRC-related surgeries over a four-year period reported that less than 1% had IBD. Those with an IBD diagnosis experienced significantly longer hospital stays and higher postoperative infection and readmission rates, as well as an increased requirement for blood transfusions, compared to non-IBD patients [95].

The surgical management of patients with IBD has significantly evolved over the past two decades. This evolution includes not only changes in surgical indications but also the introduction of innovative techniques such as robotic surgery, single-port laparoscopy, and transanal approaches, which offer potentially less-invasive alternatives [96]. 

The standard surgical approach for UC patients diagnosed with CRC or HGD is total proctocolectomy with ileal pouch-anal anastomosis (IPAA) [94]. This technique, first introduced by Parks and Nicholls in 1978, can be executed through either mucosectomy of the rectal mucosa followed by a hand-sewn anastomosis to the dentate line or via a double-stapling technique [97]. Although rectal mucosectomy is technically challenging and may negatively impact the anal transition zone, leading to worse functional outcomes [98], it significantly reduces the incidence of subsequent malignancy [24]. Conversely, the stapled anastomosis technique is generally easier to perform, allowing for a 1–2 cm “cuff” of rectal mucosa to remain. This method is associated with a lower incidence of anastomotic complications, improved bowel function, and enhanced quality of life for patients [99]. However, it is essential to recognize that stapled anastomosis carries a potential risk of adenocarcinoma developing from the residual rectal mucosa cuff [94].

Laparoscopic IPAA is a minimally invasive technique offers several advantages, including improved preservation of female fertility, reduced analgesic requirements, enhanced cosmetic outcomes, better visualization during surgery, and shorter hospital stays compared to traditional open surgery [98]. The laparoscopic approach is recommended where surgical expertise is available [100]. However, in patients with a history of previous abdominal surgeries or those with a high body mass index (BMI), a conventional surgical approach may be more appropriate [101]. 

Most patients experience a good quality of life after pouch surgery. However, long-term monitoring is crucial especially in patients with PSC-IBD and in those which the pouch was performed due to colorectal neoplasia. The overall risk of dysplasia and carcinoma in the pouch is around 1–3% [102,103]. Risk factors for dysplasia in the pouch include PSC, backwash ileitis, and rectal dysplasia at the time of IPAA, and these patients require regular (annual) endoscopic surveillance [103].

While proctocolectomy remains the most commonly performed surgical intervention for managing CRC in patients with UC, segmental resection may be considered for patients with multiple comorbidities or colonic CD with rectal sparing, provided they are in endoscopic remission [15]. An alternative to total proctocolectomy and IPAA is total colectomy with ileorectal anastomosis (IRA), which is indicated in select cases. This procedure is appropriate for patients with indeterminate colitis where CD cannot be ruled out, PSC-IBD with rectal sparing, and in cases of metastatic cancer with a limited life expectancy. Additionally, IRA may be considered for patients who have contraindications to ileostomy, such as those with ascites or portal hypertension [101].

## 9. Conclusions and Future Directions

In conclusion, patients with colonic IBD are at increased risk of dysplasia and CRC but the overall rate of colon cancer and dysplasia is decreasing. The downward trend in CRC in IBD is likely due to increased awareness, surveillance, enhanced endoscopic imaging, and better medical management of inflammation. However, certain patients with colonic IBD remain at higher risk for developing colonic dysplasia and CRC. This highlights the importance of endoscopic surveillance in all patients with colonic IBD starting at 8 years after diagnosis (or at time of diagnosis in PSC) and the interval of surveillance is subsequently determined by the overall patient risk profile. HD-WLC remains the gold standard for endoscopic surveillance of dysplasia in IBD. Dye chromoendoscopy is clearly superior to standard definition WLC but its superiority to HD-WLC remains debated. Dye chromoendoscopy is indicated in patients with PSC and those with a history of invisible dysplasia. Advancement in endoscopic techniques has offered the opportunity of non-surgical management of dysplasia. An IBD experienced multidisciplinary team approach is essential in managing patients with IBD and colorectal neoplasia. Future research is still warranted in areas of uncertainty including the optimal surveillance interval of patients with extensive pseudopolyps, surveillance of the J-pouch, Hartman’s pouch, utility of non-targeted biopsies, and advanced imaging techniques.

## Figures and Tables

**Table 1:** Selected risk factors for developing colorectal neoplasia in patients with IBD [23].

Risk Factor (Number of Studies)	HR (95% CI)
Extensive disease (2)	4.03 (0.59–27.50)
Disease Severity	
Endoscopic inflammation (1)	2.39 (1.63–3.50)
Histological inflammation (3)	2.51 (1.75–3.61)
Presence of dysplasia	
Low-grade Dysplasia (4)	3.67 (2.23–6.06)
Indefinite for Dysplasia (1)	6.85 (1.78–26.36)
Co-existing Primary Sclerosing Cholangitis (4)	2.77 (1.76–4.38)
Male Gender (7)	1.48 (1.10–1.99)
Family History of CRC (2)	2.42 (1.14–5.16)
Post-inflammatory Polyps (3)	1.73 (0.88–3.40)

CRC: colorectal cancer; HR: Hazard Ratio; CI: confidence interval.

**Table 2:** Summary of randomized controlled trials comparing high-definition white light colonoscopy to dye chromoendoscopy for the surveillance of dysplasia in inflammatory bowel disease.

Author (Publication Year)	Population	Type of Dye	Primary Endpoint Definition	Primary Endpoint Results
Mohammed et al. (2015) [56]	UC: 103	0.2% indigo carmine	Presence of dysplasia on a per-lesion and per-patient basis	DCE: 14 dysplastic lesions in 11 patients HD-WLC: 6 dysplastic lesions in 5 patients
Iacucci et al. (2017) [57]	UC: 85 (47.2%) CD: 91 (50.1%) IBD-U: 4 (2.2%)	0.04% methylene blue 0.03% of indigo carmine	Detection rates of colonic neoplastic lesions	DCE: 27 dysplastic lesions in 22 patients HD-WLC: 42 dysplastic lesions in 23 patients
Alexandersson et al. (2020) [58]	UC:173 (65.5%) CD: 87 (33%) IBD-U: 3(1%)	0.3–0.5% indigo carmine	Number of patients with ≥1 dysplastic lesion	DCE: 19 patients (29 lesions) HD-WLC: 8 (9 lesions)
Wan et al. (2021) [59]	UC: 86	0.1% methylene blue	Number of colonoscopies that diagnosed dysplasia/CRC	DCE: 14 colonoscopies HD-WLC (non-targeted biopsy): 12 colonoscopies HDWLE (targeted biopsy): 3 colonoscopies
Yang et al. (2019) [60]	UC: 210	0.05% diluted indigo carmine 0.16% diluted indigo carmine	Dysplasia detection rates	DCE: 21 patients HD-WLC: 13 patients
Feurestein et al. (2020) [61]	UC: 53 (59.6%) CD: 35 (39.3%) IBD-U: 1 (1.1%)	N/A	Detection rates of dysplastic lesions	DCE: 4 patients HD-WLC: 2 patients
Park et al. (2016) [62]	UC: 210	Indigo carmine	Detection rates of colitis-associated dysplasia	DCE: 5 patients HD-WLC: 3 patients
Groen (2024) [63]	UC: 185 (57.7%) CD: 138 (40.5%) IBD-U: 6 (1.8%)	N/A	CRN detection (proportion of procedures with macroscopic CRN)	DCE: 13.1% (28 patients) HD-WLC: 6.1% (7 patients)

UC: ulcerative colitis; CD: Crohn’s disease; IBD: inflammatory bowel disease; CRN: colorectal neoplasia; DCE: dye chromoendoscopy; HD-WLC: high-definition white light colonoscopy.
